# Review of Sensory Integration Therapy for Children With Cerebral Palsy

**DOI:** 10.7759/cureus.30714

**Published:** 2022-10-26

**Authors:** Vaishnavi B Warutkar, Rakesh Krishna Kovela

**Affiliations:** 1 Physiotherapy, Ravi Nair Physiotherapy College, Datta Meghe Institute of Medical Sciences (Deemed to be University), Wardha, IND; 2 Physiotherapy, Nitte Institute of Physiotherapy, NITTE (Deemed to be University), Mangaluru, IND

**Keywords:** gross motor function, balance, hippotherapy, sensory integration therapy (sit), neurodevelopmental therapy (ndt), cerebral palsy

## Abstract

Cerebral palsy (CP) refers to a group of non-progressive brain disorders. Several different approaches are used to treat cerebral palsy children like neurodevelopmental therapy (NDT), sensory integration therapy (SIT), and hippotherapy. Sensory integration therapy is a clinically based approach that places an emphasis on the relationship between the therapist and the child and uses play-based sensory and motor activities to encourage analysis and integration. SIT seems to offer a lot of therapeutic prospects. It uses various interventions. According to sensory integration therapists, some impacts of SIT include an improved ability to concentrate in academic, therapeutic, and social settings. Sensory integration treatment is successful in enhancing gait, balance, and gross motor function.

## Introduction and background

Cerebral Palsy (CP) is a group of permanent disorders of movement and posture causing activity limitation, which is attributed to non-progressive disturbances that occur in the developing fetal or infant's brain [1]. It influences a children's mobility, eyesight, learning, and reasoning. It can happen before, throughout, or within the first year of a child's life. It can occur up to the age of two years, as the maturation of the cortex occurs at the age of two years and CP affects the brain in an immature stage. It arises in several children as a response to cerebral hypoxia, and preterm newborns are more likely to acquire CP [1]. It is a long-term physical impairment caused by upper motor neuron damage that impacts 1.5-2.5 infants per thousand live births worldwide, resulting in a global prevalence of 17 million individuals. CP is a clinical term for children who exhibit signs of injury acquired in the course of perinatal, prenatal, or initial postnatal phases, instead of a disease manifestation in the usual term. The clinical signs of CP vary greatly depending on the kind of motor dysfunction, the amount of adaptive capability and restriction, and the afflicted parts of the body. Although there is a lack of remedies for brain damage at this time, advances are being made in both prevention and therapy [2]. Spasticity is characterized by an exaggerated stretch reflex, which becomes more prominent as the intensity of movement increases [3]. As a result, excess and inappropriate muscle activity increases, which may lead to muscle hypertonia [3]. In clinical practice, spastic motor types of CP are most commonly seen, and the consequences of spasticity include gait difficulties and exhaustion [4]. In individuals with CP, the hamstrings, rectus femoris, psoas, gastroc-soleus, and adductors are the mainly impacted lower limb muscles. The most observed spasticity in the upper extremities is in external rotators of the shoulder, elbow joint, wrist joint, finger flexors, and elbow pronators [3]. Spasticity is believed to increase calorie expenditure and impair voluntary function when moving. Joint contractures and structural deflections can change external and internal lever arms, which can cause abnormal joint forces while locomotion [3].

CP is classified according to the topographical classification (monoplegia, hemiplegia, diplegia, and tetraplegia) and the symptoms of neurological impairment (spastic, hypotonic, athetoid, or a combination of any two). Clinical symptoms varied depending on gestational age at birth, chronologic age, distribution of lesions, and underlying pathology [5].

Clinical management of children with CP aims to promote efficiency and involvement in everyday activities while eliminating the impacts of a condition that might aggravate the illness, like seizures, eating difficulties, hip dislocation, and spinal deformity such as scoliosis. Some of the therapeutic methods include improving neurological functioning since the initial stages, reducing clinical co-morbidities, weakening, and hypertonicity, using rehabilitation techniques to restore functional ability, and minimizing subsequent muscular diseases [2].

The severity of CP and its reaction to therapy can be assessed using several measures. The most broadly adopted evidence-based indicator is the Gross Motor Function Classification System (GMFCS). It is an age-based assessment that assesses gross muscle activity of children up to the age of 12 years in a variety of areas, including movement, posture, and equilibrium, and assigns a severity level to each of those areas. Level I denotes minimal restrictions (e.g., walking freely), while level V implies critical restrictions (e.g., needing a wheelchair). After being classified with GMFCS, individuals may be observed as they grow to see whether therapies result in enhancing GMFCS levels [6]. Also, the Gross Motor Function Measure-88 scale (GMFM-88), a renowned standard tool that has already been used since 1993 and has been demonstrated to be a valid and accurate assessment scale, is typically used to assess the muscle function of CP patients [7].

Different approaches used in CP

Exercise is an important approach for a child with spastic diplegic CP and is characterized as an organized, systematic, and repetitive practice that tries to enhance fitness. Because of their effects on the basic impairments of CP, aerobic and resistance exercise may enhance activity and involvement. Strength training, endurance training, and cardiovascular health are all aspects of physical fitness that exercise can help with. Resistance training is all about working or sustaining against an imposed force. The application of force is commonly done with body weight, weight training, mechanical weights, and TheraBands (TheraBand, Akron, Ohio, United States). Exercise could also improve a child with CP in contexts of pain reduction and standard of living [8].

Neurodevelopmental therapy (NDT) is the most frequently applied treatment technique for children with CP. The goal of targeting the central neurological and neuromuscular system helps the brain to increase motor efficacy and functional independence by enabling standard postural-control motions. One of the principles of NDT is task-oriented training, which has been proven to be very effective and convenient in enhancing performance by encouraging intensive, purposeful, and goal-oriented practice. The therapist's hands-on treatment of trunk activation in standing and sitting positions is included in the therapy session. Weight transfers and trunk elongation on ideal trunk alignments in standing and seated posture are several pursuits. All of these activities help children with CP improve trunk strength, balance, and gross motor coordination [9].

Children with CP benefit from dynamic surface exercise therapy as it improves trunk coordination and gross motor activity. When they work out on a dynamic interface, they get proprioceptive as well as sensory input related to the positions of respective body segments in space, and adapted motor coordination reactions to stimulus. Sensory-motor inputs can be improved by activities that take place in an unstable setting with a feedback mechanism [10]. Activities to activate muscles of the trunk on a physioball (Swiss ball) have been demonstrated to increase trunk muscular strength in healthy subjects. In a three-dimensional (3D) area, a variable field will maintain the state of arousal at the proper level. When the activities are conducted on an uneven surface instead of on a couch, the possible muscle stimulation is improved more. Because the motion of the ground underneath the individual causes postural perturbations, the muscles' response to maintain the ideal posture will be more prominent [11].

Some studies have briefly looked into the benefits of rehabilitation robots in restoring gait function in children with CP. Robot-assisted gait training (RAGT) has been proven to enhance gait velocity, gait stamina, and gross motor control in children with CP. Furthermore, most of the prior research has emphasized the results of RAGT on children with CP while incorporating stationary robotics like the Lokomat® (DIH Medical, Beijing, China), although less is featured regarding the effect of wearing exoskeleton robots. Moreover, there aren't many studies that have looked into the effects of wearable exoskeletons on gait kinetics and kinematics. Honda Walking Assist (HWA) (Honda Motor Co., Ltd, Minato City, Tokyo, Japan) is a movable exoskeleton-type device that supports hip extension as well as flexion among both limbs while locomotion. The HWA only aids one joint and does not restrict the level of flexibility in other joints, which will be enough for adequate gait learning [12].

In the therapeutic context, a variety of devices is utilized in addition to conventional therapy to assist children to strengthen their walking abilities. The treadmill is a type of device that has often been utilized for nervous system therapy. Through repetitive weight loading on a participant's lower extremities, the treadmill successfully develops gait ability. Multiple systematic reviews have reported the significant impacts of repetitive task-oriented learning employing the treadmill on motor development among individuals [13].

Hippotherapy (HPT) is an equine-assisted treatment that involves the particular movement of horses to improve neurological functions and sensory processing in individuals with neurological diseases. In recent times, research into HPT as an adjunct treatment to established therapies has expanded. HPT is focused on two basic systems that involve the conveyance of heat, and the transmission of 3D motions by periodic stimuli from the horse towards the child’s body. The child's pelvis is transferred in a manner that is regular, repetitive, and gentle, comparable to the motion that occurs in human gait. This 3D practice enhances postural balance and trunk straightening by stimulating balance responses. The alternate raising up of the horse's back, which causes anteversion/retroversion, elevation/depression, and also lateral motion with rotation, gives movements in all movement planes. HPT also delivers sensory information and promotes improved posture stability and motor coordination [14].

Sensory integration therapy (SIT)

SIT is a clinical-based technique that emphasizes the therapist-child interaction and employs play-based sensory and motor exercises to promote sensation processing and integration. SIT appears to have significant potential as a treatment [15]. Occupational therapists use this technique to assist children to develop their sensory processing and integration so that they can respond appropriately to everyday stimuli [16]. Sensory processing is receiving, trying to organize, and interpreting data via sensory inputs (e.g., touch, smell, taste, sight, hearing, and vestibular) to create an appropriate reaction. Different interventions that are used in SIT are given in Table 1 [17,18].

**Table 1:** Different interventions used in SIT SIT: sensory integration therapy

Intervention	Contents
Visual processing tasks	Block designs, matching shapes in photographs, puzzles, identifying geometric forms and alphabets, numbers, and categorization.
Body recognition	Indicating various body parts, life-size drawings, rolling right and left sides, and recognizing the body parts by touching.
Tactile awareness	sense different textures, touch boards, and recognize shapes.
Visual-motor coordination training	Ocular-pursuit training, games using pegboards and moving balls
Proprioception	Joint compression, ball squeezing, ball catching and throwing, wall push-ups

Ayres defines sensory integration as the potential to develop adequate motor and behavioral reactions to stimulus. According to her, these people had issues with registration (input detection and processing), modulation (input inhibition or transmission), associating with a few items, and/or motivation. She linked registration and modulation with two neural structures: the limbic system and the vestibular and proprioceptive systems [19]. The vestibular system processes the sensory perceptions from physical movements across space. The sensory information from muscles and joints is processed by the proprioceptive system. If it is impeded it can cause complications like hand flapping. Balls, swings, trampolines, brushes, and other devices meant to evoke proprioception, and tactile, and vestibular demands are utilized to give such sensations in playing. Deep pressure, joint compression, oral moral workouts, and body massage can all be used to increase arousal levels. Proprioceptors in muscles and joints, inner ear receptors, and auditory, visual, and tactile receptors on the skin are all triggered by activities that target many sensory systems at a time [16]. According to a new definition of SI developed from the nomenclature of sensory integration, the disorder is "difficulty detecting, modulating, interpreting, and/or responding to sensory inputs, which is severe enough to disrupt participation in everyday living activities and routines, as well as learning" [20]. Sensory integration therapists state a few effects of SIT, increased capacity to concentrate in academic, clinical, and contextual situations, decreased unwanted behavior such as self-harming actions, and better brain functionality in skills such as language and reading [21].

## Review

Methods

An electronic search was done in PubMed, Cumulated Index to Nursing and Allied Health Literature (CINAHL), Google Scholar, and Science Direct. Search terms used were sensory integration therapy, spastic diplegic CP, cerebral palsy, and different approaches used in cerebral palsy. In the same search, the Boolean terms used were “WITH”, “AND”, and “OR”.

**Figure 1:**
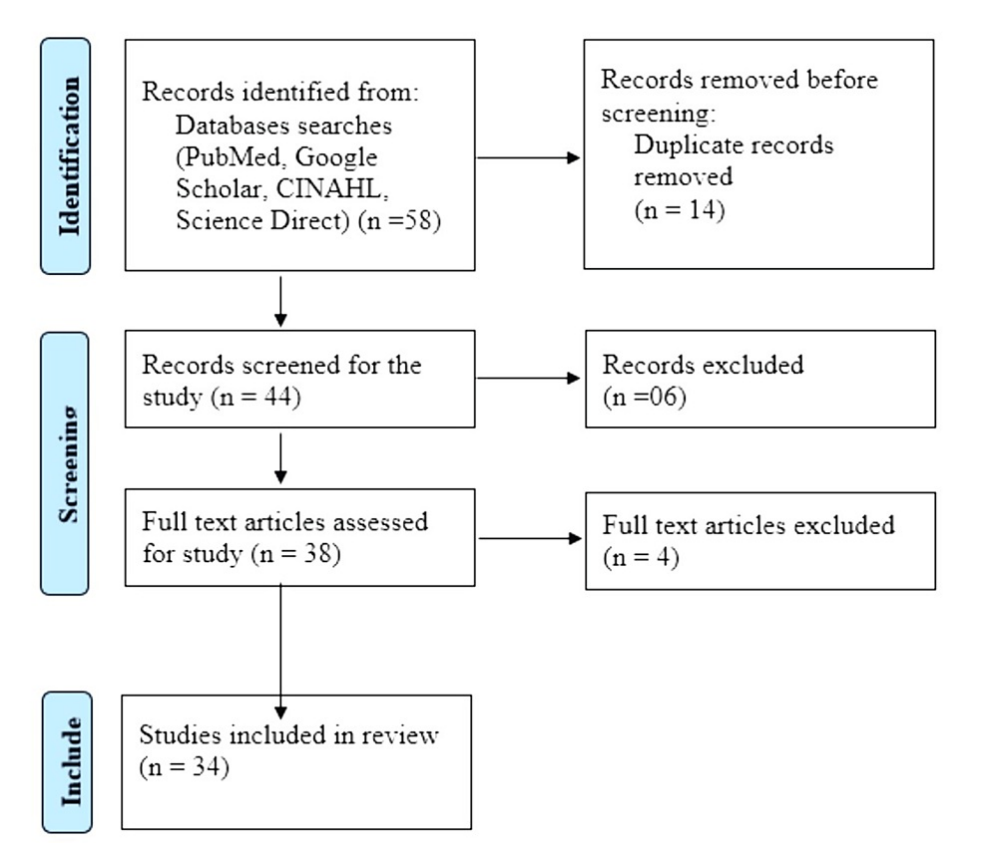
PRISMA flow diagram PRISMA: Preferred Reporting Items for Systematic Reviews and Meta-Analyses; CINAHL: Cumulated Index to Nursing and Allied Health Literature

Types of the study included literature reviews, systematic reviews, randomized controlled trials (RCTs), and experimental studies. A summary of selecting the articles has been given as per PRISMA guidelines in Figure 1.

Discussion

Effect of SIT to Improve Gross Motor Function

According to Shamsoddini and Hollisaz, the SIT approach had a considerable beneficial impact on gross motor functioning in children with CB. To enhance motor function, in each session, children were sustained on forearms and hands in sitting, quadruped, half-kneeling, and standing postures, with the OT (occupational therapist) assisting them unless tone attenuation was accomplished. After the child had achieved the ability to hold the training positions, a CP ball and tilt board were used to promote balancing and corrective reflexes. Ambulation practice (crawling, creeping, walking in a half-kneeling posture, and stepping on parallel bars) was provided at the appropriate motor stage of development [22].

A study by Mahaseth et al. states that SIT improves a children's capacity to analyze and integrate sensory data by incorporating various visual processing, kinesthetic awareness, tactile awareness, visuomotor coordination development, and vestibular and proprioceptive activities [18].

Vestibular and proprioceptive programs promote balance and awareness of position. Sensory motor development occurs throughout the first two years of a child's life, when the kid learns to integrate big muscles of the legs, trunk, with arms, as well as tiny muscles of the hands, by a variety of sensory stimuli. The outcome of sensory-motor stimulation on the gross and fine motor components is significant [23]. Sensory integration aids in the development of a mental as well as physical structure inside a person's nervous system that allows them to effectively process sensory information, manage their reactions, and comprehend the meaning of a specific texture, motion, or voice. Tahir et al. stated that in children with CP (spastic diplegia), SIT seemed to have a considerable beneficial impact on gross motor function. The exercises were created to provide active stretching, muscular strengthening, and bearing weight on the knees, feet, and hands. The body of the kid was placed into various poses, such as downward dog, wheelbarrow walking, and kids’ yoga, which provided dynamic stretches and increased bilateral synchronization [24].

Long-term therapy is required for children with CP to enhance their motor function, which includes 24-hour postural management such as special seating, night positioning, and standing frames, which is critical not just in avoiding musculoskeletal problems such as joint contractures, musculoskeletal defects, dislocations, subluxations, and reduced bone density, but also in producing a beneficial impact on the nervous system, muscle tone, spasticity, exaggerated reflexes, and kinesthetic sense [25].

Posture care is a specifically chosen treatment strategy that has a favorable influence on a child's body functioning and structure while also facilitating increased mobility and participation and accordingly improving gross motor function. As per the placement during the daytime and nighttime, since spastic cerebral palsy children have impaired joint and kinesthetic sensation, sitting, semi-reclining, and side-lying enhance oxygen saturation (SpO2) when compared to supine lying. By aiding upper limb function, appropriate sitting aids in the development of functioning in children. Feet rest, hip belts, cutout tables, and individual-based seat angles are all vital components of a good comfortable position. According to the literature, the most supportive feeding posture is an erect sitting posture with back support and head aligned with the trunk [26].

Effect of SIT on Balance and Gait

The somatosensory, vestibular, and visual systems are responsible for posture and mobility regulation. This knowledge facilitates the CNS in anticipating the forces that are delivered to the body and then generating enough muscle activation to sustain posture [27]. Conversely, each sensory input has its significance and value, and the dependability of one sort of sensory information can impact the reliability of another [28]. Vestibular stimulation generates appropriate postural reactions by altering somatosensory and visual system sensitivity, implying sensory integration [29].

The results of Seyam et al. revealed that adding SIT to children with spastic CP enhanced their gait in both spatial and temporal aspects. According to their study, Sensory integration treatment markedly enhanced gait patterns among children with hemiplegic CP [30].

Children can develop their posture stability and motor abilities by getting specialized and appropriate sensory stimuli during therapy. As a result, their interaction might be improved with the environment and social participation [31]. Pavao et al. conducted a search of the literature on sensory information alteration as well as its implication on postural stabilization in children with CP, as well as a review of comprehensive articles on the subject. Visual information as well as proprioceptive data were mentioned in the research as essential regulating processes of postural control in children with CP [32].

Research conducted by Hussein et al. says that, since the gait has recently needed different attentional resources, the children obtain proprioceptive input (motor task) plus visual feedback using appearing lights on the display (cognitive task) when walking on the Tekscan walkway platform (Tekscan, Inc., Norwood, Massachusetts, United States). As a result, it could be more efficient than typical gait training in terms of improving spatial and temporal gait variables [33].

A summary of a few articles is given in Table 2.

**Table 2:** Summary of articles included in the study BADL: Barthel activities of daily life; GMFCS: gross motor function classification system; MAS: modified Ashworth scale; GMFM-88: gross motor function measure-88; TOA-NDT: task-oriented activities based on neurodevelopmental therapy; PBS: pediatric balance scale; SIT: sensory integration therapy; CP: cerebral palsy

Author and Year	Design	Sample size	Intervention	Outcome measure	Conclusion
Gillani et al., 2021 [1].	Prospective case series	134 males and 66 females	Conservative and surgical treatment methods.	BADL, GMFCS level I-IV, MAS	The final grading scale of therapy on CP children ranges from fair to good outcomes, with both therapies (conservative and surgical) having a favorable impact.
Bar On et al., 205 [3].	Review				They came to the conclusion that it is incorrect to classify all resistance to passive motion as spasticity and that hypertonia should be seen as complex.
Reid et al [4].	Cross-sectional study	264 children		GMFCS, the Viking Speech Scale, Manual Ability Classification System, The Eating and Drinking Ability Classification System, and Communication Function Classification System.	According to the study, including useful tools in clinical settings regularly will enable precise and reliable categorization of primary and secondary motor types, topographic, and functionality skills.
Sharova et al., 2018 [7].	Comparative study	23 spastic CP children – study group 15 healthy children – control group	Rehabilitation exercise program	GMFM-88 Scale, GMFCS	According to research, physical therapy and rehabilitation services that emphasize neural plasticity can help children with cerebral palsy lower their immune system's cellular and humoral reactions.
Ryan et al., 2017 [8].	Review	29 trials			Before making any strong inferences about the efficacy of exercise for persons with CP, it is necessary to conduct enormous, exceptional, very well-reported randomized trials that evaluate the efficiency of training with respect to both participation and activities.
Sah et al., 2019 [9].	Randomized clinical trial	44 spastic diplegic CP children	task-oriented activities based on neurodevelopmental therapy (TOA-NDT), traditional physiotherapy	PBS, trunk impairment scale (TIS), Gross motor function measure-88 (GMFM-88), and postural assessment scale (PAS).	postural control, stability, and gross motor coordination can be greatly improved by using TOA-NDT principles.
Schindl et al., 2000 [10].	Open, non-randomized, baseline-treatment study.	10 children	Treadmill training	Functional ambulation categories, GMFM-88	In children who are unable to walk, treadmill exercise with partial body weight bearing is a potential therapy method.
Reddy and Balaji [11].	Randomized controlled trial	30 children	dynamic surface exercise training (DSET), standard physiotherapy	GMFM-88 and PBS	Combining the two had a positive impact on trunk stability and gross motor skills.
Kawasaki et al [12].	A pilot randomized controlled trial	10 spastic CP children	Robot-assisted gait training (RAGT) and non-assisted gait training (NAGT) were carried out on the treadmill along with the Honda Walking Assist (HWA)	GMFCS	The findings imply that augmenting both hip motions with the honda walking assist may enhance gait in children with CP
Guindos-Sanchez et al., 2020 [14].	Review	10 studies (452 participants)	Hippotherapy	GMFM-88	Gross motor control in individuals with CP was improved by Hippotherapy.
Mahaseth and Choudhary, 2021 [18].	Comparative randomized controlled trial	30 children	Conventional physical therapy and sensory integration therapy	GMFM, Short Sensory Profile (SSP)	Combining both proved to be more productive and efficient in increasing gross motor skills in CP children.
Shamsoddini and Hollisaz, 2009 [22].		24 diplegic spastic CP children	SIT, a home program	GMFM-88	Research demonstrated more advantages of the SIT training plan for individuals with CP.
Rauf et al., 2021 [26].	Experimental study	74 children	body positioning and postural techniques	GMFM-88 and Modified Ashworth Scale	All five domains of functioning were enhanced by proper body alignment and postural methods.

## Conclusions

CP is a common disorder of development and movement that affects a lot of children and they present with a gamut of deficits therefore they should be rectified with the use of an appropriate strategy of treatment. Improving their activities of daily living and functionality is always a challenge. Treatments for children with CP are beneficial in several ways. SIT though not new but an important one. Sensory integration treatment focuses on a few distinct areas of rehabilitation, such as vision, touch, and proprioception. SIT is also asserted to be successful in enhancing gait, balance, and gross motor functioning. As a result, it could be useful in learning new motor skills.
